# The Role of the Vaginal Microbiome in Preterm Premature Rupture of Membranes: A Comprehensive Review of Mechanisms and Clinical Implications

**DOI:** 10.1002/hsr2.71484

**Published:** 2025-12-14

**Authors:** Maryam Alikamali, Sakineh Mohammad‐Alizadeh‐Charandabi, Somayeh Ahmadi, Mohammad Yousef Memar, Mahnaz Shahnazi

**Affiliations:** ^1^ Student Research Committee, Faculty of Nursing and Midwifery Tabriz University of Medical Sciences Tabriz Iran; ^2^ Faculty of Nursing and Midwifery, Social Determinants of Health Research Center Tabriz University of Medical Sciences Tabriz Iran; ^3^ Infectious and Tropical Diseases Research Center Tabriz University of Medical Sciences Tabriz Iran; ^4^ Department of Midwifery, Faculty of Nursing and Midwifery Tabriz University of Medical Sciences Tabriz Iran

**Keywords:** dysbiosis, inflammation, PPROM, treatment, vaginal microbiome

## Abstract

**Background and Aims:**

Preterm premature rupture of membranes (PPROM), a complication in approximately 4.5% of pregnancies, is a leading cause of preterm birth and significant perinatal morbidity. A substantial body of evidence implicates vaginal dysbiosis a departure from a healthy, *Lactobacillus*‐dominant microbiome in the pathogenesis of PPROM. This review synthesizes the current understanding of the mechanistic links between the vaginal microbiome and PPROM and discusses the clinical implications for future therapeutic strategies.

**Methods:**

A comprehensive literature search was conducted in the PubMed, Scopus, and Google Scholar databases. The selection focused on peer‐reviewed articles including systematic reviews, meta‐analyses, clinical trials, and influential observational studies (e.g., cohort and case‐control), as well as key preclinical studies investigating the vaginal microbiome, PPROM pathogenesis, and relevant therapies.

**Results:**

The synthesized evidence supports a multi‐step mechanistic framework wherein ascending pathobionts, characteristic of dysbiosis, trigger a host inflammatory cascade via Toll‐like receptors. This inflammatory milieu orchestrates a synergistic attack on fetal membrane integrity through three primary pathways: (1) enzymatic degradation of the extracellular matrix by matrix metalloproteinases (MMPs), (2) programmed cell death (apoptosis) of membrane cells, and (3) damage from oxidative stress. Although conventional therapies such as antibiotics have limitations, emerging strategies, including probiotics, immunomodulators, and antioxidants, are being developed to target these specific mechanistic pathways.

**Conclusion:**

This review positions the vaginal microbiome as a central player in the pathophysiology of PPROM, rather than merely a risk factor. This mechanistic understanding shifts the therapeutic focus from broad‐spectrum antibiotics toward targeted therapies designed to prevent dysbiosis or neutralize specific downstream inflammatory and degradative pathways. Translating this knowledge into effective clinical practice through rigorous randomized controlled trials remains a critical priority for improving perinatal outcomes.

## 1 Introduction

Premature rupture of membranes (PROM) is defined as the rupture of the amniotic sac before the onset of labor. This event is termed preterm premature rupture of membranes (PPROM) when it occurs before 37 weeks of gestation [1]. PPROM complicates approximately 4.5% of pregnancies and contributes to one‐third of all preterm births [2]. Adverse outcomes of PPROM are numerous and include early‐onset neonatal sepsis, chorioamnionitis, fetal and maternal death (with an estimated maternal mortality rate of 4.5 per 10,000 in pre‐viable cases), cord compression, asphyxia, placental abruption, and postpartum endometritis [3, 4, 5]. Membrane weakening and subsequent rupture stem from a range of factors, including elevated local cytokines, an imbalance between matrix metalloproteinases (MMPs) and their tissue inhibitors, and heightened collagenase and protease activity, which can increase intrauterine pressure [6]. Intra‐amniotic infection is a common comorbidity of PPROM [7]. The risk factors for PPROM are multifactorial and include a history of PPROM, a short cervical length, vaginal bleeding in the second or third trimester, uterine overdistention, nutritional deficiencies in copper and vitamin C, connective tissue disorders, a low body mass index, lower socioeconomic status, and smoking [8, 9]. Among these factors, imbalances in the vaginal microbiota—termed vaginal dysbiosis—have emerged as a key contributor to the pathogenesis of PPROM. This relationship is mediated by complex interactions involving the host immune system, inflammatory processes, and the structural integrity of the fetal membranes [10]. The role of the microbiome and metabolites produced by microbes present in different parts of the body has been shown to play in the development of various diseases [11]. In a healthy pregnancy, the vaginal microbiome is dominated by *Lactobacillus* species. These bacteria produce lactic acid to maintain an acidic vaginal environment, which is critical for inhibiting the proliferation of pathogenic microorganisms [12]. Vaginal dysbiosis occurs when protective *Lactobacillus* species decline, allowing for the overgrowth of pathogenic bacteria such as *Gardnerella vaginalis* and *Ureaplasma urealyticum* [10]. This microbial shift promotes local inflammation, weakens the fetal membranes, and increases the risk of ascending infection, collectively culminating in premature membrane rupture [13]. Indeed, studies reveal that women with PPROM often exhibit distinct microbial patterns, characterized by higher bacterial diversity and an abundance of species associated with bacterial vaginosis (BV) [14, 15]. Therefore, elucidating the mechanisms linking microbial imbalance to membrane rupture is crucial for developing targeted therapies to mitigate PPROM risk. This review provides an in‐depth examination of these mechanisms, exploring how dysbiosis contributes to membrane rupture and infection, and discusses the implications for prevention and treatment.

### 1.1 Search Strategy and Selection Criteria

This narrative review synthesizes literature identified through a comprehensive search of the PubMed, Scopus, and Google Scholar databases for articles published up to May 2025. The search strategy used a combination of keywords and MeSH terms, including “PPROM,” “vaginal microbiome,” “dysbiosis,” “inflammation,” and “treatment.” The selection process prioritized peer‐reviewed, English‐language articles, including systematic reviews, meta‐analyses, clinical trials, and influential observational studies (e.g., cohort, case‐control), along with key preclinical investigations. We generally excluded case reports and conference abstracts unless they offered unique mechanistic insights.

## 2 The Vaginal Microbiome and PPROM

### 2.1 The Vaginal Microbiome: Composition, Function, and Importance in Pregnancy

The vaginal microbiome comprises a complex community of microorganisms, predominantly bacteria, but also including fungi and viruses. A healthy vaginal microbiome is typically characterized by the dominance of *Lactobacillus* species, such as *L. crispatus*, *L. jensenii*, *L. gasseri*, and *L. iners*. These beneficial bacteria play a critical role in maintaining vaginal health by producing lactic acid, which sustains an acidic pH (3.8–4.5). This acidic environment is fundamental to host defense, as it inhibits the proliferation of pathogenic microorganisms [16, 17]. Furthermore, some *Lactobacillus* species produce antimicrobial compounds like hydrogen peroxide (H₂O₂) and bacteriocins, providing an additional layer of protection [18, 19]. The composition of the vaginal microbiome is highly dynamic and influenced by numerous factors, including hormonal fluctuations (e.g., menstrual cycle, pregnancy, menopause), sexual activity, and the use of antibiotics or contraceptives [20]. During pregnancy, elevated estrogen levels increase glycogen deposition in the vaginal epithelium. This glycogen serves as a primary nutrient for lactobacilli, promoting their growth and reinforcing a stable, protective microbiome [21]. Consequently, the vaginal microbiome in most healthy pregnancies shifts toward a stable, low‐diversity state dominated by *Lactobacillus* [22, 23]. This state is considered protective against ascending infections that can lead to adverse pregnancy outcomes. However, a significant subset of pregnant individuals develops vaginal dysbiosis, characterized by a depletion of lactobacilli and an overgrowth of anaerobic bacteria like *G. vaginalis*, *Atopobium vaginae*, and *Prevotella* species [24].

This condition, often diagnosed as BV, is a major risk factor for preterm birth, miscarriage, and chorioamnionitis [25]. Emerging evidence suggests that the vaginal microbiome profile in early pregnancy may predict later outcomes. For instance, lower *Lactobacillus* dominance and higher instability during the first trimester have been associated with subsequent preterm delivery [26]. Specific bacteria, such as *Mycoplasma hominis* and *A. vaginae*, have been linked to local inflammatory responses that can weaken fetal membranes by promoting collagen degradation, predisposing them to rupture [27, 28, 29]. Moreover, 16S rRNA gene sequencing studies have confirmed that a high‐diversity microbiome with a prevalence of anaerobes like *A. vaginae* and *Sneathia* species in the second trimester is associated with an increased risk of PPROM, likely due to their capacity to trigger pro‐inflammatory pathways [30, 31, 32]. Table 1 summarizes key evidence linking the vaginal microbiome to PPROM.

**Table 1:** Summary of studies investigating the association between the vaginal microbiome and PPROM.

Authors	Country (year)	Type of study	Case (*N*, age)	Control (*N*, age)	Inclusion criteria	Intervention	Sample	Analysis methods	Main findings	References
Minkoff et al.	United States (1984)	Prospective study	*N* = 40	*N* = 148	13.8 ± 3.6 weeks' gestation	—	Vaginal swabs	Cultures	↑*Trichomonas vaginalis*, *Bacteroides* sp.	[33]
Mcdonald et al.	Australia (1992)	Descriptive prospective study	*N* = 57	*N* = 651	Before 37 completed weeks gestation	—	Vaginal swabs	Cultures	*↑U. urealyticum*, *Bacteroides* sp.	[34]
Grattard et al.	France (1995)	Retrospective study	*N* = 208	*N* = 208	Before 37 completed weeks gestation	—	Cervical vaginal swabs	PCR	*↑Ureaplasma urealyticum, Mycoplasma hominis*	[35]
Nasution et al.	Malaysia (2007)	Retrospective study	*N* = 48	*N* = 48	Before 37 weeks gestation	—	Vaginal swab	PCR	*↑Ureaplasma urealyticum, Mycoplasma hominis*	[36]
Kacerovský et al.	Czech (2009)	Case control study	*N* = 225	*N* = 225	24 and 36 weeks	—	Cervical swabs	Cultures	*↑Ureaplasma urealyticum, Mycoplasma hominis*	[37]
Baldwin et al.	USA (2015)	Cohort study	*N* = 15 29 years ±4	*N* = 5 29 years ±4	< 34 weeks gestation	Antibiotic treatment (ampicillin/amoxicillin and azithromycin, clindamycin, azithromycin)	Dacron swabs	MiSeq. 600 cycle v3 kit (Illumina, San Diego, CA)	*↓Weeksella*, *Lachnospira*, *Achromobacter*, and *Pediococcus* ↑*Prevotella*, *Peptoniphilus*, *Peptostreptococcus*, and *Tissierellaceae* ph2 The relative abundance of Lactobacillus, Prevotella, and Peptoniphilus was not substantially impacted during the hospitalization of the PPROM subjects	[38]
Lee et al.	Korea (2016)	Retrospective study	*N* = 199 15−50	*N* = 313 15−50	Severe pathological conditions	—	Vaginal swabs	Culture	*↑U. urealyticum*	[39]
Jayaprakash et al.	Canada (2016)	Prospective cohort study	*N* = 36 32.92 ± 4.83	—	28.8 week	—	Vaginal swabs	PCR	↑*Megasphaera* type 1 and *Prevotella* spp.	[40]
Brown et al.	UK (2018)	Prospective cohort study	*N* = 87 33 (32–34)	—	Before 37 weeks gestation	Erythromycin	Vaginal swabs	PCR Illumina MiSeq platform (Illumina Inc)	*↓Lactobacillales* ↑ *Sneathia* spp.	[15]
Saghafi et al.	Iran (2018)	Cohort study	*N* = 200	—	27−37 weeks	Azithromycin, ampicillin	Endocervical canal swabs	Cultures	*Escherichia coli* (24.2%), Coagulase‐negative Staphylococci (27.2%), *Enterococcus* and *Candida* each 1 (11.7%)	[41]
Brown et al.	UK (2019)	Prospective cohort study	*N* = 60 33.8 (31.2–34.3)	*N* = 36 Matched by case (normal pregnancy controls)	Before 37 weeks gestation	—	Vaginal swabs	PCR, Illumina MiSeq platform (Illumina Inc.)	*↓Lactobacillales* ↑*Prevotella*, *Peptoniphilus*, and *Dialister*	[14]
Liu et al.	China (2021)	Retrospective study	*N* = 36 29.1 (22–39) (selected six cases with PROM metagenomics)	*N* = 87 29.5 (19–40) (selected five HCs for metagenomics)	Retrospectively pregnant women that have some risk factor for PPROM	—	Vaginal swabs	PCR, DNA nanoball sequencing method, Metagenemark	↓L*actobacillus crispatus*, *Lactobacillus iners*, *Lactobacillus gasseri*, and *Lactobacillus jensenii* ↑*Streptococcus*, *Chlamydia*, *Prevotella*, *Staphylococcus*, *Mycobacterium*, and *Enterobacter*	[48]
Elshabrawy et al.	Egypt (2021)	Case control study	*N* = 320 28.7 ± 9.03	*N* = 320 29.9 ± 10.8	28−37 weeks of gestation	—	Vaginal swabs	Smear, cultures	*↑Streptococcus agalactiae*	[49]
Liu et al.	China (2022)	Prospective study	*N* = 45 28.96 ± 3.80	*N* = 90 29.70 ± 3.66	The third trimester of pregnancy	—	Vaginal swabs	Illumina MiSeq	*↓Lactobacillus* spp. *↑Gardnerella, Prevotella*, *Megasphaera*, *Ureaplasma*, and *Dialister*, *Aerococcus, Arcanobacterium*, *Veillonellales_Selenomonadales, Mycoplasmatales, Bacteroidales, Bifidobacteriales, Lachnospiraceae, Eggerthellaceae, Aerococcaceae, Aerococcus, Romboutsia, Klebsiella*	[50]
Gulav et al.	Kenya (2022)	Case control study	*N* = 49 32.7 ± 5.10	*N* = 49 31.5 ± 4.50	Between 26 and 36 6/7 weeks gestation	Antibiotics given more than 24 h before enrollment or within the last 4 weeks	Swabbing the posterior vaginal fornix	Illumina MiSeq, QIIME2	*↑L. iners, L. crispatus, L. jensenii, Gardnerella*, *Prevotella*	[51]
Yan et al.	China (2022)	Cross‐sectional study	*N* = 48 31.0 ± 5.03	*N* = 54 29.67 ± 3.71	Between 24 and 36 + 6 weeks	—	Vaginal swabs	Illumina NovaSeq PE250 platform	*↓Lactobacillus crispatus, Lactobacillus gasseri* ↑*Lactobacillus iners*, *Gardnerella vaginalis*, *Prevotella bivia*, *Ochrobactrum* sp., *Prevotella timonensis*, *Ureaplasma parvum*	[31]
Mu et al.	China (2023)	Case‐control study	*N* = 29 28.63 (24.63−32.63)	*N* = 180 27.75 (24.53−30.97)	Pregnant females in the early second trimester	—	Vaginal swabs	Ion S5 XL instrument, QIIME 2 software	*↑L. iners, L. paragasseri/gasseri, Atopobium vaginae, Gardnerella vaginalis*	[52]
Mikula et al.	Austria (2023)	Cohort study	*N* = 24	—	Before 29 gestational weeks	Ampicillin, cefazoline, azithromycin	Vaginal swabs	Culture	*↓Beta‐hemolytic streptococci group B and Gardnerella vaginalis*	[53]
Borges et al.	Germany (2023)	Prospective multicenter cohort study	*N* = 78	—	Hospitalization between 22 and 34 weeks of gestation	Aminopenicillin	Vaginal samples	Illumina MiSeq sequencing	*↓Lactobacillus* spp *↑Ureaplasma parvum*	[54]

*Note:* The table summarizes findings from selected prospective, retrospective, case‐control, and cohort studies. All listed microbial findings are in comparison to the control group or baseline.

Abbreviations: N, number of participants; PCR, polymerase chain reaction; ↑, increased abundance or positive association; ↓, decreased abundance or negative association.

### 2.2 Vaginal Dysbiosis and Its Clinical Implications

Vaginal dysbiosis represents a significant departure from the healthy, *Lactobacillus*‐dominated state (eubiosis). This state is defined by a marked depletion of protective *Lactobacillus* species and a corresponding overgrowth of diverse anaerobic bacteria, including pathobionts such as *G. vaginalis*, *Prevotella* species, and *A. vaginae* [10]. This dysbiotic profile is often classified as community state type IV (CST‐IV), a state characterized by high microbial diversity and a low abundance of *lactobacilli* [42]. The clinical implications of dysbiosis are profound, frequently manifesting as BV, the most common vaginal condition in women of reproductive age. More critically, vaginal dysbiosis is strongly associated with adverse reproductive and obstetric outcomes, including an increased risk of sexually transmitted infections (STIs), pelvic inflammatory disease (PID), miscarriage, and, central to this review, chorioamnionitis and PPROM, which is a leading cause of preterm birth [10, 42].

### 2.3 Describe the Mechanisms Linking the Vaginal Microbiome to PPROM

**Figure 1:**
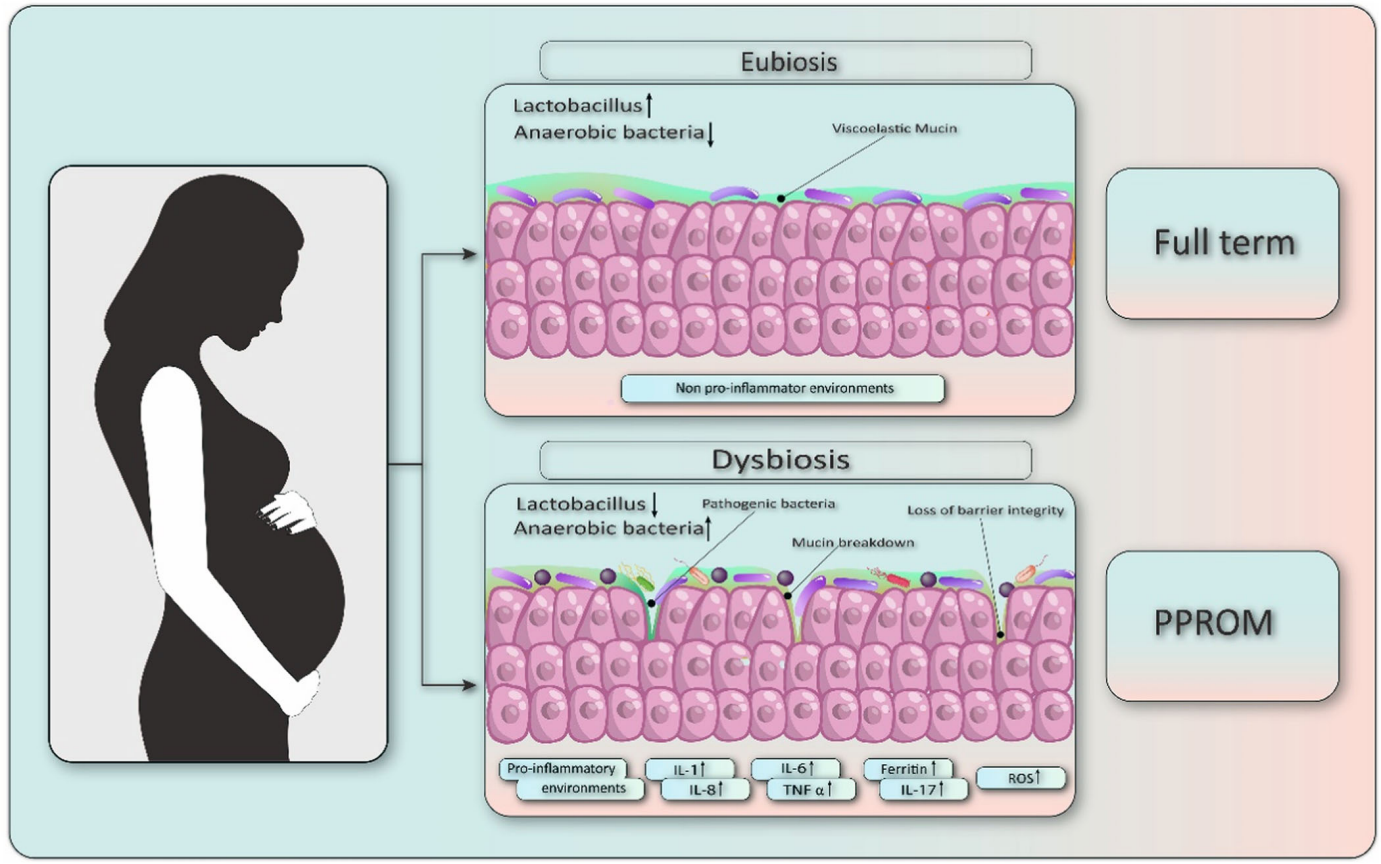
Proposed mechanisms linking the vaginal microbiome to pregnancy outcomes. The figure illustrates the contrast between vaginal eubiosis and dysbiosis during pregnancy. (Top panel) Eubiosis: A healthy vaginal microbiome, characterized by a high abundance of *Lactobacillus* species (↑) and low abundance of anaerobic bacteria (↓), maintains a protective layer of viscoelastic mucin and a non‐pro‐inflammatory environment, which is associated with a full‐term pregnancy. (Bottom panel) Dysbiosis: A shift in the microbiome, characterized by a decrease in *Lactobacillus* (↓) and an increase in pathogenic anaerobic bacteria (↑), leads to mucin breakdown and a loss of barrier integrity. This disruption triggers a pro‐inflammatory environment, marked by the release of cytokines (e.g., IL‐1β, IL‐6, IL‐8, IL‐17, TNF‐α), ferritin, and reactive oxygen species (ROS), which weakens the fetal membranes and contributes to PPROM.

The mechanistic framework linking vaginal microbiome alterations to PPROM comprises a multi‐step inflammatory and structural failure cascade. Vaginal dysbiosis initiates this process, wherein a loss of protective *Lactobacillus* allows for the ascension of pathobionts. In the upper reproductive tract, Toll‐like receptors (TLRs) on chorioamniotic and cervical cells recognize pathogen‐associated molecular patterns (PAMPs), triggering a potent innate immune response and a subsequent cascade of pro‐inflammatory cytokines [43]. Figure 1 visually summarizes this proposed mechanistic cascade.

Direct biochemical evidence supports the components of this framework. For instance, the resulting inflammatory milieu orchestrates membrane weakening through three primary, synergistic pathways. First, through enzymatic degradation, key inflammatory instigators like TNF‐α and IL‐1β directly stimulate amniochorionic cells to produce and activate MMPs. Biochemical studies using zymography have confirmed significantly elevated MMP‐9 activity in the fetal membranes of women with PPROM [44, 45]. Second, inflammatory signals and immune cells induce programmed cell death (apoptosis) in membrane cells; this mechanism is validated by TUNEL assays, which demonstrate a significantly higher apoptotic index in PPROM cases [46]. Third, the inflammatory response generates high levels of reactive oxygen species (ROS), causing oxidative stress, a finding confirmed by measuring elevated markers of oxidative damage in patients with PPROM [47].

The culmination of this multi‐pronged, biochemically validated attack results in a critical loss of biomechanical integrity, rendering the membranes fragile and susceptible to premature rupture.

### 2.4 Controversies and Contradictory Findings

Although a general link between vaginal dysbiosis and PPROM is well‐supported, the role of specific microorganisms remains debated, with contradictory findings in the literature. *Ureaplasma* and *Mycoplasma* species serve as a prime example. Numerous studies have associated the presence of these microbes in the lower genital tract with an increased risk of PPROM and preterm birth [55]. However, other studies have failed to find a significant association. These organisms are also found in a substantial proportion of uncomplicated term pregnancies, suggesting they may act as commensals in some contexts and become pathogenic only under certain host or environmental conditions [56].

This discrepancy likely stems from several factors. First, methodological inconsistencies, such as using culture‐based versus high‐sensitivity molecular techniques, can lead to different detection rates and biased conclusions [57]. Second, host‐specific factors, including genetic background and immune response, may determine whether colonization leads to a pathogenic outcome. Finally, virulence may be strain‐specific, making the simple detection of a species an insufficient predictor of pathology [41]. Therefore, future research must critically assess these confounding variables to clarify the true causative agents of PPROM.

### 2.5 Advances in Microbial Profiling in PPROM Research

Recent research utilizing advanced molecular techniques, such as 16S rRNA gene sequencing, has provided new insights into the vaginal microbiome of women experiencing PPROM [25, 31]. These studies employ analytical methods ranging from traditional cultures to advanced next‐generation sequencing platforms like Illumina MiSeq/NovaSeq and Ion S5 (Table 1), allowing for a more comprehensive characterization of the microbial landscape. A key application of 16S rRNA sequencing in this context is the detection of subclinical infections that may contribute to PPROM. The technique has proven invaluable for identifying fastidious or slow‐growing pathogens often missed by conventional culture methods, such as *Ureaplasma* and *Mycoplasma* species [31, 58]. By providing quicker and more thorough results than traditional cultures, 16S rRNA sequencing enables clinicians to make more timely and informed decisions, significantly enhancing patient care [59].

## 3 Treatment of PPROM

### 3.1 Antibiotics in PPROM Management

Antibiotic therapy is a cornerstone of PPROM management, primarily aimed at prolonging the latency period and reducing maternal and neonatal infectious morbidity. The landmark ORACLE I trial heavily influenced the standard of care by demonstrating that a 10‐day course of erythromycin improved key neonatal outcomes [60]. However, the optimal regimen, timing, and microbial targets remain subjects of ongoing debate. Current guidelines, such as those from the American College of Obstetricians and Gynecologists (ACOG), typically recommend a 7‐day course combining intravenous ampicillin and erythromycin, followed by oral amoxicillin and erythromycin [61]. Nevertheless, many centers now substitute azithromycin for erythromycin due to better gastrointestinal tolerance and a simpler dosing schedule, although direct comparative evidence in PPROM is limited.

A major limitation of current strategies is their broad‐spectrum nature. This empiric approach does not specifically target the pathobionts associated with dysbiosis (e.g., *Ureaplasma*, *Gardnerella*) and can negatively impact the beneficial *Lactobacillus* population. For example, some evidence suggests that erythromycin may exacerbate the underlying dysbiosis even while prolonging latency [15]. This highlights a critical need for future research to move from broad‐spectrum prophylaxis toward more precise, microbiome‐informed antibiotic therapies. Such an approach would aim to target specific pathogens identified through rapid molecular diagnostics while preserving the commensal flora essential for a healthy pregnancy.

As summarized in Table 1, clinical trials involving women with PPROM have utilized a range of antibiotic regimens, including erythromycin, ampicillin, and azithromycin, reflecting the diverse therapeutic approaches to managing the condition.

### 3.2 Probiotics in PPROM Management

Given that vaginal dysbiosis is a key upstream trigger in the PPROM cascade, modulating the microbiome with probiotics represents a rational and highly promising preventive strategy. The theoretical goal of probiotic therapy is to restore a *Lactobacillus*‐dominant state, thereby reinforcing the vaginal epithelial barrier, lowering pH, and outcompeting potential pathogens [62, 63]. However, despite this strong biological rationale, the clinical evidence supporting the efficacy of probiotics for the specific prevention of PPROM remains speculative and insufficient. Major systematic reviews and meta‐analyses, including a comprehensive Cochrane review, have concluded that there is currently no high‐quality evidence to suggest that probiotic supplementation during pregnancy significantly reduces the risk of PPROM or other preterm birth outcomes [64, 65]. The available trials are often limited by small sample sizes and significant heterogeneity in the probiotic strains, doses, and timing of intervention, making firm conclusions difficult [66]. Therefore, while probiotics are generally considered safe in pregnancy, their role in PPROM prevention is currently investigational. Large, well‐designed randomized controlled trials (RCTs) that target high‐risk women with specific, well‐characterized probiotic strains and clearly defined protocols are urgently needed to translate the promising mechanistic concepts into effective clinical practice.

### 3.3 Tocolytics in PPROM Management

The use of tocolytics in the management of PPROM remains a subject of considerable debate [67]. While clinicians routinely employ these agents to suppress uterine contractions in preterm labor [68], their role in PPROM is primarily to delay delivery for a short, critical period. This brief delay aims to permit the administration of antenatal corticosteroids for fetal lung maturation or to facilitate maternal transfer to a center with a neonatal intensive care unit (NICU), thereby improving neonatal survival and reducing morbidity [69]. Commonly employed tocolytics in PPROM include magnesium sulfate, calcium channel blockers (e.g., nifedipine), β2‐adrenergic agonists (e.g., terbutaline), NSAIDs (e.g., indomethacin), and oxytocin receptor antagonists (e.g., atosiban) [68]. However, their use is tempered by the primary concern of infection. Membrane rupture creates a portal of entry for bacteria, and prolonging the latency period with tocolytics may inadvertently increase the risk of chorioamnionitis, as well as maternal and neonatal sepsis [70]. Despite these risks, selective use of tocolysis can be beneficial. The ACOG guidelines permit a short course (up to 48 h) of tocolytics before 34 weeks of gestation to allow corticosteroids to take effect. This strategy is associated with reduced rates of respiratory distress syndrome and neonatal mortality. The decision to initiate tocolysis must be individualized, weighing gestational age against the risks of infection and other contraindications [69, 71]. Limited data suggest progesterone and hydroxyprogesterone caproate may have a role in maintenance tocolysis, but they are not considered primary tocolytic agents [68]. However, the inconsistent evidence regarding improved long‐term outcomes emphasizes the importance of conducting further research into alternative therapies and interventions. Ultimately, as a systematic review points out, while tocolysis can prolong latency, its impact on key perinatal outcomes remains uncertain [72]. This inconsistent evidence emphasizes the need for further research into alternative therapies and interventions [73].

### 3.4 Amnioinfusion in the Management of PPROM Management

Amnioinfusion, a procedure originally developed to alleviate umbilical cord compression during labor, has been investigated as a therapeutic strategy for PPROM [74]. This procedure involves infusing a sterile solution into the amniotic cavity to restore fluid volume. The goal is to mitigate risks associated with oligohydramnios, such as umbilical cord compression and pulmonary hypoplasia. Performed either transcervically or transabdominally, amnioinfusion aims to preserve the amniotic cushion, thereby prolonging the pregnancy and improving neonatal outcomes [75, 76]. Evidence suggests that amnioinfusion may be particularly beneficial in early PPROM [75, 77]. For instance, Kohari et al. demonstrated that serial amnioinfusions before 26 weeks of gestation significantly reduced the incidence of pulmonary hypoplasia and improved neonatal survival rates [78]. Furthermore, a Cochrane systematic review reported improved neonatal respiratory outcomes and decreased NICU admissions following the procedure [79]. Recent studies have highlighted the potential of combining continuous amnioinfusion with targeted antibiotics, such as meropenem [80]. Meropenem is an effective antimicrobial agent for complicates infections particular caused by Gram‐negative bacteria [81, 82]. This combination may enhance treatment efficacy against infections caused by resistant bacteria [80]. Despite these potential benefits, concerns remain regarding procedural risks, notably chorioamnionitis [83]. Current ACOG guidelines do not endorse routine amnioinfusion for PPROM but consider it a potential option in cases of severe oligohydramnios before fetal viability [84]. Therefore, the procedure requires meticulous monitoring to balance the potential benefits against the associated risks [79].

### 3.5 Emerging Therapeutic Approaches

In addition to conventional therapies, several investigational strategies are being explored to prevent PPROM and improve neonatal outcomes. Immunomodulatory therapies, such as monoclonal antibodies against TNF‐α and IL‐1β, have shown promise in reducing intrauterine inflammation and prolonging gestation [85, 86]. Antioxidant therapies, including N‐acetylcysteine (NAC) and melatonin, reduce oxidative damage in fetal membranes and may limit apoptosis and weakening [87]. Furthermore, targeting the degradation of the extracellular matrix is another key strategy. Therapies that directly inhibit MMP activity to preserve collagen integrity have shown encouraging results in preclinical models [88]. Finally, surgical interventions like cervical cerclage can reinforce cervical structure and reduce membrane stress, especially when combined with anti‐inflammatory or hormonal therapies [89].

## 4 Future Directions and Implementation Challenges

### 4.1 Structured Future Research Directions

To translate the mechanistic understanding of the microbiome's role in PPROM into effective clinical strategies, future research must be structured and multi‐pronged. First, in basic and translational science, there is a need to move beyond 16S rRNA profiling to multi‐omics approaches (metagenomics, transcriptomics, metabolomics). This will help elucidate not just which microbes are present, but what metabolic and functional pathways are active during dysbiosis‐driven membrane weakening [90, 91]. Second, preclinical animal models remain crucial for testing the efficacy and safety of novel targeted therapies, such as specific MMP inhibitors or immunomodulators, before human trials. Third, future RCTs must be designed with greater precision. They should target well‐defined, high‐risk populations (e.g., women with a history of PPROM and a confirmed dysbiotic profile), and interventions must specify the exact probiotic strains, dose, and, critically, the timing of initiation, with a focus on early pregnancy to target the “window of opportunity” before significant inflammation is established [88].

### 4.2 Practical Implementation Challenges

Even with positive trial results, translating these findings into routine clinical practice presents significant challenges. A major hurdle is the lack of rapid, cost‐effective screening tools to identify at‐risk women based on their vaginal microbiome profile early in pregnancy. Furthermore, the standardization of interventions, particularly with probiotics, is a major issue. Probiotics are often regulated as supplements, not drugs, leading to wide variability in strain composition, dosage, and quality control, which complicates the development of evidence‐based clinical guidelines [92]. Finally, the optimal timing of intervention may be in the first trimester, a period where many women have limited contact with prenatal care, posing a significant logistical challenge for implementation.

## 5 Conclusions

This review synthesizes the evidence that repositions the PPROM‐associated vaginal microbiome from a simple risk factor to a central player in a distinct pathophysiological cascade. The evidence supports a framework where dysbiosis, characterized by a depletion of protective *Lactobacillus* and an overgrowth of pathobionts, initiates an ascending inflammatory process mediated by host pattern recognition receptors. This inflammation does not merely associate with PPROM; it appears to directly contribute to it by orchestrating a synergistic attack on fetal membrane integrity through MMP‐driven enzymatic degradation, widespread cellular apoptosis, and oxidative stress. The clinical implications of this mechanistic understanding are significant. Moving beyond simple antibiotic prophylaxis, future therapeutic strategies should focus on either preventing the initial dysbiosis or targeting these specific downstream pathways. Interventions such as targeted probiotics, immunomodulators, and antioxidants represent rational, mechanism‐based approaches to prevent PPROM. Translating this mechanistic understanding into effective clinical practice through rigorous RCTs remains a critical priority for improving perinatal outcomes [43, 85, 93].

### 5.1 Limitations

Although this review summarizes recent progress in understanding the vaginal microbiome's role in PPROM, there are still several limitations to consider. First, much of the existing evidence comes from observational studies, which cannot definitively prove a direct causal link between dysbiosis and membrane rupture. Moreover, the studies use different methods for microbial profiling, which makes it difficult to compare their findings. Finally, this review did not delve deeply into significant nonmicrobial factors influencing PPROM.

For instance, genetic predisposition can play a significant role, with certain polymorphisms in genes related to inflammation (e.g., TNF‐α, IL‐6) and collagen metabolism (e.g., MMPs) being associated with an increased susceptibility to PPROM [94, 95]. Similarly, environmental factors, including exposure to air pollutants such as particulate matter and maternal lifestyle choices like smoking, have been identified as important contributors that were not the focus of this microbial‐centric review [96]. A further significant limitation, as highlighted by the heterogeneity of the studies summarized in Table 1, is the inconsistency in microbial profiling methods used across the literature. The included studies employed a range of techniques, from traditional culture‐based approaches to various next‐generation 16S rRNA sequencing platforms. These methods have fundamentally different detection thresholds and inherent biases; culture‐based methods can only identify viable, culturable microorganisms, whereas sequencing‐based techniques detect a much broader spectrum of microbial DNA but can be influenced by factors such as primer selection and sequencing depth [42]. This methodological heterogeneity makes direct comparison of microbial prevalence and abundance across studies challenging and may contribute to some of the varied findings in the field [97]. Consequently, while this review synthesizes the available evidence, conclusions must be interpreted with caution, and standardization of methods is a critical need for future research.

Furthermore, this review discusses the vaginal microbiome in broad terms and does not fully address the significant interindividual and population‐level variability that exists. Seminal studies have established that the composition of the vaginal microbiome and the prevalence of different community state types (CSTs) vary significantly across women of different ethnic and geographic backgrounds [98]. For instance, a *Lactobacillus*‐depleted, high‐diversity state (CST‐IV), which is strongly associated with dysbiosis, is more prevalent in women of African and Hispanic descent compared to Caucasian and Asian women [10, 98]. This suggests that what constitutes a “healthy” or “high‐risk” microbial profile may not be universal. This variability, driven by host genetics, diet, and other environmental factors, likely influences an individual's susceptibility to PPROM and their response to potential therapies. Therefore, the conclusions drawn from studies conducted in one population may not be directly generalizable to others, a critical consideration for both future research and clinical practice.

## Author Contributions

**Maryam Alikamali:** methodology, writing – original draft, writing – review and editing, investigation. **Sakineh Mohammad‑Alizadeh‑Charandabi:** writing – review and editing, writing – original draft, methodology, investigation. **Somayeh Ahmadi:** methodology, writing – original draft, writing – review and editing, software. **Mohammad Yousef Memar:** conceptualization, methodology, writing – review and editing, writing – original draft, validation, project administration. **Mahnaz Shahnazi:** writing – review and editing, writing – original draft, conceptualization, supervision.

## Ethics Statement

The authors have nothing to report.

## Conflicts of Interest

The authors declare no conflicts of interest.

## Transparency Statement

The lead author, Mohammad Yousef Memar, and Mahnaz Shahnazi, affirm that this manuscript is an honest, accurate, and transparent account of the study being reported; that no important aspects of the study have been omitted; and that any discrepancies from the study as planned (and, if relevant, registered) have been explained.

## Data Availability

Data sharing is not applicable to this article as no data sets were generated or analyzed during the current study.
